# The Diagnostic Journey of a Patient with Prader–Willi-Like Syndrome and a Unique Homozygous *SNURF-SNRPN* Variant; Bio-Molecular Analysis and Review of the Literature

**DOI:** 10.3390/genes12060875

**Published:** 2021-06-07

**Authors:** Karlijn Pellikaan, Geeske M. van Woerden, Lotte Kleinendorst, Anna G. W. Rosenberg, Bernhard Horsthemke, Christian Grosser, Laura J. C. M. van Zutven, Elisabeth F. C. van Rossum, Aart J. van der Lely, James L. Resnick, Hennie T. Brüggenwirth, Mieke M. van Haelst, Laura C. G. de Graaff

**Affiliations:** 1 Department of Internal Medicine, Division of Endocrinology, Erasmus MC, University Medical Centre Rotterdam, 3015 GD Rotterdam, The Netherlands; k.pellikaan@erasmusmc.nl (K.P.); a.rosenberg@erasmusmc.nl (A.G.W.R.); e.vanrossum@erasmusmc.nl (E.F.C.v.R.); a.vanderlelij@erasmusmc.nl (A.J.v.d.L.); 2 Dutch Centre of Reference for Prader-Willi Syndrome, 3015 GD Rotterdam, The Netherlands; 3 Department of Neuroscience, Erasmus University Medical Centre, 3015 GD Rotterdam, The Netherlands; g.vanwoerden@erasmusmc.nl; 4 The ENCORE Expertise Centre for Neurodevelopmental Disorders, Erasmus University Medical Centre, 3015 GD Rotterdam, The Netherlands; 5 Department of Clinical Genetics, Erasmus University Medical Centre, 3015 GD Rotterdam, The Netherlands; l.vanzutven@erasmusmc.nl (L.J.C.M.v.Z.); h.bruggenwirth@erasmusmc.nl (H.T.B.); 6 Department of Clinical Genetics, Amsterdam UMC, University of Amsterdam, 1081 HV Amsterdam, The Netherlands; l.kleinendorst@amsterdamumc.nl (L.K.); m.vanhaelst@amsterdamumc.nl (M.M.v.H.); 7 Institute of Human Genetics, University Hospital Essen, University Duisburg-Essen, 45147 Essen, Germany; bernhard.horsthemke@uni-due.de (B.H.); christian.grosser@humangenetik-tuebingen.de (C.G.); 8 Praxis für Humangenetik Tübingen, 72076 Tuebingen, Germany; 9 Obesity Center CGG, Erasmus MC, University Medical Centre Rotterdam, 3015 GD Rotterdam, The Netherlands; 10 Department of Molecular Genetics and Microbiology, College of Medicine, University of Florida, Gainesville, FL 32610, USA; jresnick@UFL.EDU; 11 Academic Centre for Growth Disorders, Erasmus MC Rotterdam, 3015 GD Rotterdam, The Netherlands

**Keywords:** prader–willi syndrome, genetic variation, genomic imprinting, genetics, brain

## Abstract

Prader–Willi syndrome (PWS) is a rare genetic condition characterized by hypotonia, intellectual disability, and hypothalamic dysfunction, causing pituitary hormone deficiencies and hyperphagia, ultimately leading to obesity. PWS is most often caused by the loss of expression of a cluster of genes on chromosome 15q11.2-13. Patients with Prader–Willi-like syndrome (PWLS) display features of the PWS phenotype without a classical PWS genetic defect. We describe a 46-year-old patient with PWLS, including hypotonia, intellectual disability, hyperphagia, and pituitary hormone deficiencies. Routine genetic tests for PWS were normal, but a homozygous missense variant NM_003097.3(*SNRPN*):c.193C>T, p.(Arg65Trp) was identified. Single nucleotide polymorphism array showed several large regions of homozygosity, caused by high-grade consanguinity between the parents. Our functional analysis, the ‘Pipeline for Rapid in silico, in vivo, in vitro Screening of Mutations’ (PR*i*SM) screen, showed that overexpression of *SNRPN-p.Arg65Trp* had a dominant negative effect, strongly suggesting pathogenicity. However, it could not be confirmed that the variant was responsible for the phenotype of the patient. In conclusion, we present a unique homozygous missense variant in *SNURF-SNRPN* in a patient with PWLS. We describe the diagnostic trajectory of this patient and the possible contributors to her phenotype in light of the current literature on the genotype–phenotype relationship in PWS.

## 1. Introduction

Prader–Willi syndrome (PWS) is a rare genetic condition (estimated prevalence of 1:10,000–1:30,000), affecting multiple organ systems [1]. In the neonatal period, PWS is characterized by muscular hypotonia and feeding difficulties, which usually require tube feeding. Later in infancy, patients switch to hyperphagia (overeating) due to abnormal satiety response to food intake [1,2,3]. Endocrine features include growth hormone deficiency, hypothyroidism, hypogonadism, and, rarely, central adrenal insufficiency [1,3,4]. Both hyperphagia and pituitary dysfunction contribute to abnormal weight gain, which can ultimately lead to obesity. Additionally, temperature regulation and pain registration are often disturbed [1,2,5]. The majority of these features can be explained by hypothalamic dysfunction [6]. Moreover, patients with PWS often display developmental delay, autism spectrum disorders (ASD), ASD-like behavior [1,7], and challenging behavior [3]. The physical appearance is characterized by short stature, obesity, almond-shaped eyes, strabismus, small bitemporal diameter, a thin upper lip, small hands and feet, and tapering fingers [1,3]. Commonly used consensus diagnostic criteria are those proposed by Holm et al. [8] in 1993 (Table 1).

PWS is usually caused by the absence of expression of a cluster of paternally expressed genes on chromosome 15q11.2-q13. The maternal allele is imprinted, and therefore genomic and epigenetic changes lead to PWS only if they occur in the paternally expressed genes. PWS is most commonly caused by a paternal deletion (70–75%), a uniparental maternal disomy 15 (mUPD, 25–30%), an imprinting center defect (ICD, 1–3%), or a paternal chromosomal translocation (rare) [9,10]. The so called ‘Prader–Willi syndrome critical region’ on chromosome 15q11.2-q13 encompasses several genes, including *MKRN3*, *MAGEL2*, *NDN, NPAP1*, *SNURF-SNRPN,* and numerous non-coding RNAs (ncRNAs) including small nucleolar RNAs (*snoRNAs*) [10]. The exact function of most of these genes and their exact relation to the PWS phenotype still remains unclear. 

In patients with Prader–Willi-like syndrome (PWLS), PWS features are present without the classical PWS genotype (paternal deletion, mUPD, ICD, or translocation of chromosome 15q11.2-13). To provide adequate treatment and genetic counselling to the patients with PWLS and their relatives, it is important to understand the underlying genetic defects and pathways [10]. Cases with PWLS provide novel insight into the complex genotype–phenotype relationship of PWS. Many chromosomal alterations involving different chromosomes have been described in relation to PWLS [10,11,12]. Several cases of PWLS with a small deletion in the PWS critical region have been described. Deletions ranged from 80 to 236 kb and most include *SNORD116* and the ‘imprinted in Prader–Willi syndrome’ (*IPW*) gene [13,14,15,16,17,18,19]. These case reports suggest that the region encoding *SNORD116* and *IPW* is responsible for the key characteristics of PWS, while other genes in the PWS critical region may have smaller phenotypic contributions.

We now present a patient with PWLS, with a normal result on genetic diagnostic testing for PWS, without abnormalities in *SNORD116* or *IPW*. We describe the fascinating diagnostic trajectory and discuss possible explanations for her phenotype in light of the current knowledge on the genotype–phenotype relationship in PWS.

## 2. Materials and Methods

The patient and her mother, who is legal representative, gave permission for the use of the clinical information and pictures in the current paper.

### 2.1. Methylation-Specific Multiplex-Ligation Probe Amplification (MS-MPLA)

For the MS-MLPA, we used the Salsa MLPA ME028-B2 PWS/AS probemix (MRC-Holland©), as described by Beygo et al. [20].

### 2.2. Single Nucleotide Polymorphism (SNP) Array

Genomic DNA was extracted from peripheral blood and hybridized to a Human CytoSNP-12 array (Illumina, San Diego, CA, USA) as reported before [21]. The array was scanned with the Illumina iScan Control. Data were processed using Genome Studio v2.1 software and analyzed with Nexus Copy Number software v5.0 (Biodiscovery, El Segundo, CA, USA).

### 2.3. Next Generation Sequencing (NSG)

Obesity gene panel sequencing was performed at the genetic diagnostics laboratory of the UMC Utrecht to search for pathogenic abnormalities in the exons of 57 protein coding obesity-associated genes, as described previously [22]. Identified variants were classified according to the American College of Medical Genetics and Genomics guidelines for variant classification.

### 2.4. RNA Sequencing

A punch biopsy of the skin was taken from the index case and the mother. RNA sequencing was performed at the Institute of Human Genetics, University Hospital Essen. In order to understand whether, and how, the new variant affects gene expression, we compared RNA sequencing data from the patient and her mother to healthy controls and patients with genetically confirmed classical PWS.

### 2.5. Effect Predictor Programs

To predict whether the variant could affect protein function, we used Alamut VISUAL PLUS ^TM^ v.2.15 to access multiple protein prediction programs: Align-GVGD [23], MutationTaster (Build NCBI37/Ensembl 69) [24], sorting intolerant from tolerant (SIFT) [25,26], and Polymorphism Phenotyping v2 (Polyphen-2) [27,28].

### 2.6. PRiSM Screen

We assessed the effect of the *SNRPN* variant on protein function and its pathogenicity using our in-house developed functional genomics screen “Pipeline for Rapid in silico, in vivo, in vitro Screening of Mutations (PR*i*SM)” (for other studies where this has been used see [29,30,31,32]).

#### 2.6.1. Constructs

To obtain the cDNA sequence from human *SNRPN* (NM_003097.6) a PCR (Phusion high fidelity, Thermo Fisher) was done on the human brain cDNA library, using the following primers: Fw 5′ GGCGCGCCACCATGACTGTTGGCAAGAGTAG 3′ and Rev 5′ TTAATTAACTAAGGTCTTGGTGGACG 3′. The gene was then cloned into our dual promoter expression vector [31,32,33]. Using PCR (Phusion high fidelity, Thermo Fisher) we then introduced the single nucleotide variant using the following primers: SNRPN– c.193c>t (p.Arg65Trp), Fw 5′ CCAGAGCGTGAAGAAAAGTGGGTTTTGGGTCTGGTGT 3′ and Rev 5′ ACAC-CAGACCCAAAACCCACTTTTCTTCACGCTCTGG 3′. The same expression vector without an inserted gene was used as control for all the in vivo and in vitro experiments (control vector).

#### 2.6.2. Mice

FvB/NHsD females were crossed with FvB/NHsD males (ordered at 8–10 weeks old from Envigo) for the neuronal cultures, whereas for the in utero electroporation female, FvB/NHsD (Envigo) were crossed with male C57Bl6/J (ordered at 8–10 weeks old from Charles River). All mice were kept group-housed in IVC cages (Sealsafe 1145T, Tecniplast) with bedding material (Lignocel BK 8/15 from Rettenmayer) on a 12/12 h light/dark cycle in 21 °C (±1 °C), humidity at 40–70%. Food pellets (801727CRM(P) from Special Dietary Service) and water were available ad libitum. All animal experiments were conducted in accordance with the European Commission Council Directive 2010/63/EU (CCD approval AVD101002017893).

#### 2.6.3. HEK-293T Cell Transfections

We cultured HEK-293T cells (not authenticated) in 6-well plates in DMEM/10% Fetal Calf Serum (FCS)/1% penicillin/streptomycin and transfected them when they were 60% confluent with the empty vector control, *SNRPN-WT* or *SNRPN-p.Arg65Trp* (3 μg per 6-well dish), using polyethylenimine (PEI) according to the manufacturer instructions (Sigma). Then, 4–6 h after transfection, we changed the medium to reduce toxicity.

#### 2.6.4. Western Blot

Two to three days after the HEK-293T cells were transfected, they were harvested and homogenized in lysis buffer (10 mM Tris-HCl 6.8, 2.5% SDS, 2 mM EDTA with added protease inhibitor cocktail (#P8340, Sigma), phosphatase inhibitor cocktail 2 (#P5726, Sigma), and phosphatase inhibitor cocktail 3 (#P0044, Sigma)). The BCA protein assay kit (Pierce) was used to determine the protein concentration. The lysate concentrations were adjusted to 1 mg/mL. Primary antibodies used: SNRPN (#11070-1-AP, 1:1000, ProteinTech) and RFP (#600401379, 1:2000, Rockland; used to detect tdTomato); secondary antibody: goat anti-rabbit (#926-68021, 1:15,000, LI-COR). LI-COR Odyssey Scanner and Odyssey 3.0 software were used to quantify the blots. The intensity of the SNRPN protein band in the different conditions was normalized against tdTomato (RFP signal). For the analysis, 4 replicates were used.

#### 2.6.5. Primary Hippocampal Cultures

We prepared the primary hippocampal neuronal cultures from FvB/NHsD wild-type mice following the previously described procedure [34]. Briefly, hippocampi from brains of E16.5 embryos were collected in 10 mL ice cold neurobasal medium (NB, Gibco). After incubation of the hippocampi in pre-warmed trypsin/EDTA solution (Invitrogen) at 37° for 20 min, they were dissociated in 1.5 mL NB medium supplemented with 2% B27, 1% penicillin/streptomycin, and 1% glutamax (Invitrogen). Neurons were then plated on poly-D-lysine (25 mg/mL, Sigma) coated coverslips (1*10^6^ cells per coverslip) in 12 well plates containing 1 mL of supplemented NB for each coverslip. The plates were stored at 37°/5% CO_2_.

#### 2.6.6. Neuronal Transfection and Immunocytochemistry

After three days in vitro (DIV), neurons were transfected with an empty vector control (1.8 μg per coverslip), *SNRPN-WT,* or *SNRPN-p.Arg65Trp* (2.5 μg per coverslip). For transfection, Lipofectamine was used according to the manufacturer’s instructions (Invitrogen). Five days post-transfection, neurons were fixed with 4% paraformaldehyde (PFA)/10% sucrose, for the neuronal morphology analysis. Antibody staining was done overnight at 4 °C with MAP2 (1:500, #188004, Synaptic System) and SNRPN (#11070-1-AP, 1:100, ProteinTech) in GDB buffer (0.2% BSA, 0.8 M NaCl, 0.5% Triton X-100, 30 mM phosphate buffer, pH 7.4). Secondary antibodies: anti-guinea-pig-Alexa647 (#706-605-148) and anti-rabbit-Alexa488 (#711-545-152) conjugated secondary antibody (1:200, Jackson ImmunoResearch). Mowiol-DABCO (Sigma) mounting medium was used to mount the coverslips. The LSM700 was used to acquire confocal images.

At least 10 confocal images (20× objective, 0.5 zoom, 1024 × 1024 pixels) of different transfected neurons (identified by the red staining from the tdTomato) were obtained from each condition, with at least two independent experimental replications. Total neurite length and arborization (the number of branching of each primary neurite) was analyzed, using the NeuronJ plugin of ImageJ. All values were normalized against the mean values of the empty vector control. Analysis was done by an experimenter blinded for the transfection conditions.

#### 2.6.7. In Utero Electroporation

The in utero electroporation was done as described previously [32]. In short, pregnant FvB/NHsD mice at E14.5 of gestation were anesthetized, and the uterus was exposed. Through the uterus wall, the DNA construct (1.5–3 μg/μL, diluted in fast green (0.05%)) was injected in the lateral ventricle of the embryos, using a glass pipette controlled by a Picospritzer^®^ III device. Using tweezer-type electrodes connected to a pulse generator (ECM 830, BTX Harvard Apparatus), five electrical square pulses of 45 V (50 ms per pulse and 150 ms inter-pulse interval (ipi)) were delivered. The positive pole of the tweezers was placed on top of the developing somatosensory cortex. Plasmids injected: empty vector control, *SNRPN-WT,* or *SNRPN-p.Arg65Trp*. After birth, pups (M/F) were sacrificed at P1 for histochemical processing.

#### 2.6.8. Immunohistochemistry

Mice (deeply anesthetized with an overdose of Nembutal) underwent transcardial perfusion with 4% paraformaldehyde (PFA) and the brains were post-fixed in 4% PFA. After embedding the brains in gelatin and cryoprotecting them in 30% sucrose in 0.1 M phosphate buffer (PB) for 2–4 h, they were frozen on dry ice, and sectioned coronally using a freezing microtome (50 μm thick). Free-floating sections were blocked in PBS containing 10% normal horse serum (NHS) and 0.5% Triton X-100 for one hour and then incubated with primary antibody RFP (#600401379, 1:2000, Rockland) in PBS containing 2% NHS, 0.5% Triton X-100, at ambient temperature overnight. Secondary antibody used: Cy3 donkey-anti-rabbit (1:400, Jackson ImmunoResearch) diluted in PBS containing 2% NHS, 0.5% Triton-X 100. 4′,6-diamidino-2-phenylindole solution (DAPI, 1:10,000, Invitrogen) was used as a counterstain and Mowiol was used to mount the sections on glass. Images were acquired using a LSM700 confocal microscope (Zeiss) with a 10× objective.

Confocal images (10× objective, 0.5 zoom, 1024 × 1024 pixels) obtained from 2–3 non-consecutive sections from at least 3 successfully targeted animals per condition were used for the neuronal migration analysis (previously described [31,32,33]).

#### 2.6.9. Statistical Analysis

We assumed normally distributed data. For the in vitro and in vivo overexpression experiments, statistical difference was determined using one-way analysis of variance (ANOVA) followed by Dunnett’s post-hoc test for multiple comparisons. Two-tailed unpaired *t*-test (dual comparison) was used for the Western blot analysis. We analyzed neuronal migration based on the proportion of electroporated cells that migrated to the cortical plate at P1 (defined as the most proximal 40% of dorsoventral distance between the pia and ventricle (first four of ten equally spaced bins)).

### 2.7. Literature Review

We performed a search on Embase, Medline, the Web of Science Core Collection, Cochrane Central Register of Controlled Trials, and Google Scholar for case reports of patients with genetic alterations (e.g., translocations, deletions, genetic variants) in the PWS critical region on the paternal chromosome that affected only part of the PWS critical region. We included case reports and case series that provide a description of the genotype and phenotype of each individual case. We included articles about patients with and without features of PWS. Exclusion criteria were articles that were not available online, articles that were not available in English, patients with genetic alterations of the PWS critical region on the maternal chromosome only, case reports where the entire PWS critical region or the PWS-imprinting center (IC) was affected, case reports where the affected region extended beyond the PWS critical region, and case reports with insufficient genotyping or phenotyping. For the full search strategy, see Table S2.

## 3. Results

### 3.1. Patient Description

The female patient presented at our hospital at the age of 46 years, after she had been referred for treatment of morbid-obesity. She fulfilled the criteria for the clinical diagnosis of PWS, as is shown in Table 1.

#### 3.1.1. Medical History

There were little fetal movements during pregnancy. The index case was born after a pregnancy of 40 weeks with a birth weight of three kilograms (−0.5 SD). Postnatally, she was cyanotic and hypotonic. Her neonatal feeding difficulties required tube feeding for which she was hospitalized for several months. Her feeding difficulties persisted until she was 1.5 years old, after which she developed hyperphagia, resulting in obesity at the age of four. When she was twelve years old, her weight was 125 kg according to her mother. Secondary to her obesity, she had developed type 2 diabetes mellitus and dyslipidaemia, for which she was treated with metformin and simvastatin, respectively. Psychomotor development was severely delayed; she started walking at the age of eight years and talking at the age of 40 years. She received special education. She had primary amenorrhea.

#### 3.1.2. Anamnesis

The patient had clear hyperphagia: She could easily eat four full plates of food. Her mother, who was the primary caregiver, reported challenging behavior including temper tantrums, stealing food, skin picking, and trichotillomania.

#### 3.1.3. Family History

The parents of the index case were first-degree relatives. The mother of the index case had many (half-) siblings, of which most were illiterate or had great difficulty reading and writing, mainly caused by lack of education during childhood. Although both the mother of the index case and multiple siblings had short stature and were overweight, none of the family members had the same phenotype as the index case. However, the mother had lost contact with most relatives and, therefore, family health history was incomplete. The mother of the patient was 157 cm tall and weighed 91 kg (body mass index (BMI) 37 kg/m^2^).

#### 3.1.4. Physical Examination

**Figure 1:**
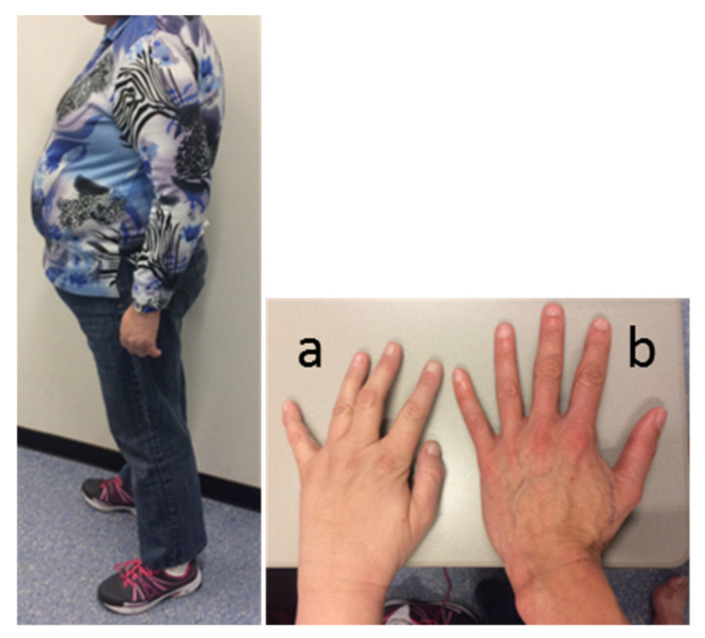
Appearance of the index case. Picture of the patient’s body, and left hand (**a**) compared to the hand of a healthy female control (**b**).

Physical examination revealed a typical PWS appearance of the index case, with obesity (BMI 34 kg/m^2^), hypotonia, kyphosis, high-arched feet (requiring orthopedic shoes), and small hands with tapering fingers. Facial features included narrow bitemporal diameter, almond-shaped eyes, and strabismus (Figure 1). She had some breast formation, but exact Tanner stage for breast development was hard to assess due to her obesity. She had Tanner stage 3 pubic hair development. Her height was 158 cm (−2 SD, [35]), her weight 85 kg, and her head circumference 56.5 cm (+0.7 SD).

#### 3.1.5. Biochemistry and Imaging

Laboratory results revealed central hypogonadism and a lowered Insulin-like growth factor (IGF)1 level (Table S1). Peak growth hormone value during growth hormone releasing hormone (GHRH) and arginine stimulation test was 2.9 µg/L. This is lower than the cut-off of 4.2 µg/L in obese subjects [36], confirming the diagnosis growth hormone deficiency. Cortisol and thyroid hormone levels were normal. Dual-energy X-ray absorptiometry (DEXA) scan showed osteopenia of the lumbar vertebrae and osteoporosis of the femoral neck. 

#### 3.1.6. Polysomnography

Polysomnography showed mild sleep apnea with an apnea–hypopnea index of 6.2 per hour [37].

#### 3.1.7. Features Not Corresponding to PWS

Apart from the typical PWS features, the patient also had features not corresponding to PWS, like celiac disease, tinnitus, hearing loss (requiring a hearing aid), cataract (requiring lens implantation), and arthralgia of the hands and feet. Atypical findings during physical examination were a remarkable overbite, diastemata, multiple naevi, and a small palpebral fissure width. Her relatively tall stature (158 cm, while most PWS females have a height below 150 cm) was also atypical for PWS. Additionally, MRI of the brain also revealed abnormalities not specific for PWS, including septo-optic dysplasia (SOD), with several midline defects, including partial agenesis of the corpus callosum and the septum pellucidum and an atrophic left optic nerve, but the pituitary gland had a normal size [38]. In addition, there was partial agenesis of the sagittal sinus and most likely agenesis of the falx cerebri. Lastly, there was a small lesion in the left-posterior part of the pituitary gland with a maximum diameter of 5 mm, most likely a pituitary incidentaloma. Her clinical, behavioral, and dysmorphic features are shown in Table 2.

**Figure 2:**
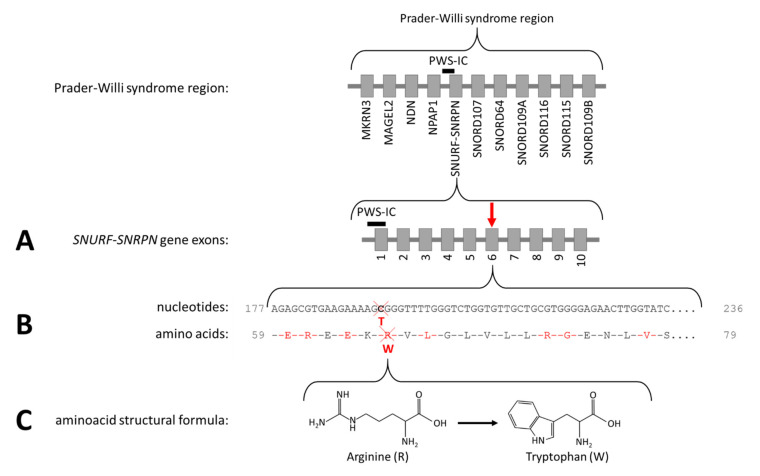
The Prader–Willi syndrome region and NM_003097.3(*SNRPN*):c.193C>T, p.(Arg65Trp). (**A**) The arrow indicates the location of the variant in the *SNURF-SNRPN* gene. (**B**) The cross indicates the location of the affected nucleotide and the amino acid. (**C**) The structural formula of arginine and tryptophan.

**Figure 3:**
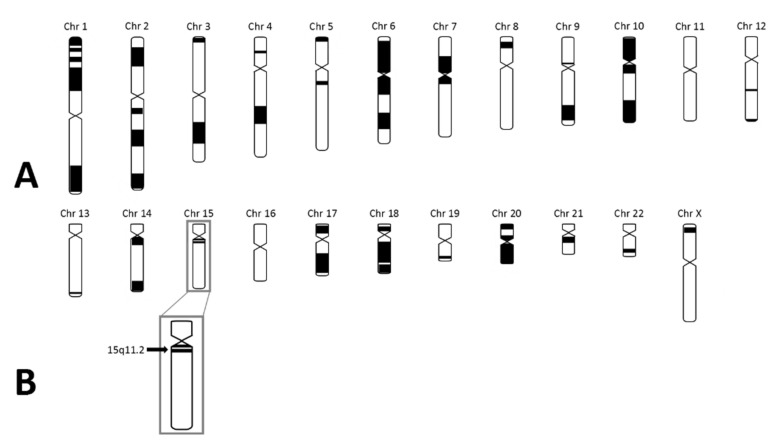
SNP array results in the index case. (**A**) Black parts represent homozygous regions in the index case. The number of homozygous regions is suggestive for high-grade consanguinity. (**B**) The black arrow represents 15q11.2 in which *SNURF-SNRPN* is located. As shown, *SNURF-SNRPN* lies within a homozygous region.

As the patient fulfilled the consensus criteria for Prader–Willi syndrome (Table 1), we performed genetic diagnostic testing for PWS. After routine PWS methylation tests, using methylation-specific multiplex-ligation probe amplification (MS-MLPA), ruled out the presence of a deletion, mUPD, or ICD of chromosome 15q11.2-13, we performed additional genetic testing. Obesity gene panel analysis revealed a homozygous variant NM_003097.3(SNRPN):c.193C>T, p.(Arg65Trp) in *SNURF-SNRPN* (Figure 2), which was classified as a variant of uncertain clinical significance (class 3 according to the American College of Medical Genetics and Genomics guidelines). This variant was mentioned in the supplementary table (individual 111) of the article by Kleinendorst et al. [22]. The mother was heterozygous for the same variant. The father was not available for DNA analysis. Hemizygosity in the index patient was excluded by MS-MLPA analysis. Single nucleotide polymorphism array (SNP) array showed several large regions of homozygosity (ROH), caused by high-grade consanguinity between the parents (Figure 3). The exact coordinates of the breakpoints can be found in Table S3.

**Figure 4:**
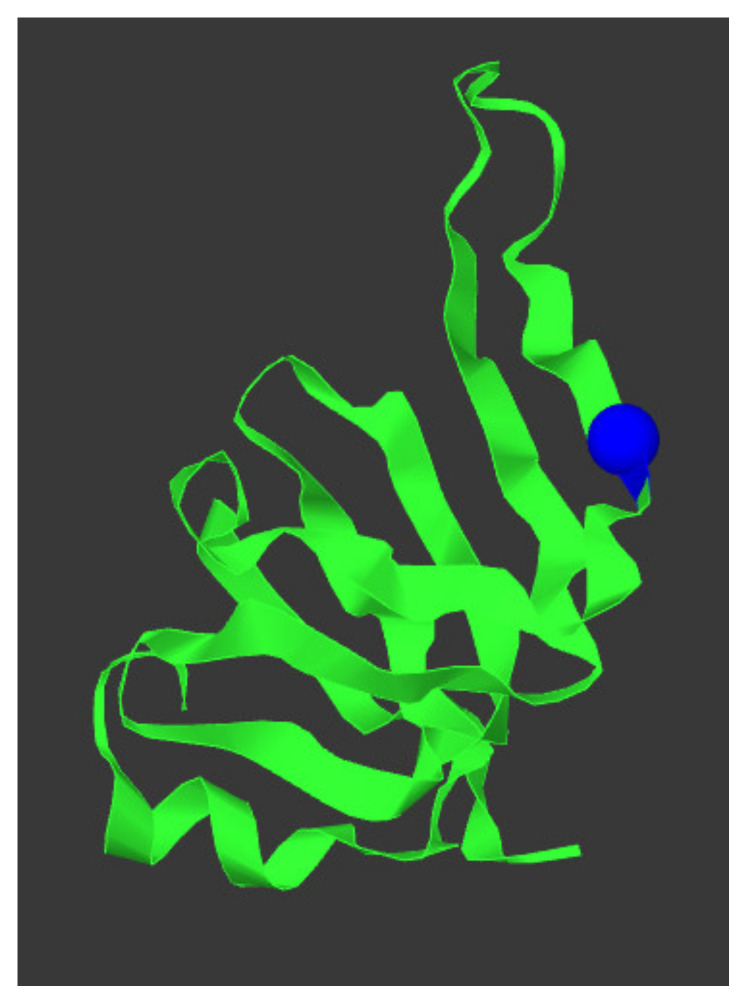
SNRPN protein structure and location p.(Arg65Trp) variant. The protein structure of SNPRN is given in green, the p.(Arg65Trp) variant is depicted as a blue sphere. This figure was generated using mutation3D [44].

*SNURF-SNRPN* was located in one of the ROH. In silico analysis using variant effect predictor programs Align-GVGD [23], MutationTaster (Build NCBI37/Ensembl 69) [24], sorting intolerant from tolerant (SIFT) [25,26], and Polymorphism Phenotyping v2 (Polyphen-2) [27,28] predicted that NM_003097.3(*SNRPN*):c.193C>T, p.(Arg65Trp) is deleterious. The variant was not present in the 1000 Genomes Project (Phase 3 release) [42] or in the genome aggregation database (gnomAD v2.1.1) [43]. The protein structure of SNRPN and the location of the changed amino acid is depicted in Figure 4. RNA sequencing analysis showed equal expression of *SNRPN* and all other genes in the PWS critical region for the index case, the mother, and four healthy, Caucasian volunteers (one male and three females) that served as controls. RNA sequencing confirmed that the mother of the patient had the variant on her paternal chromosome 15.

**Figure 5:**
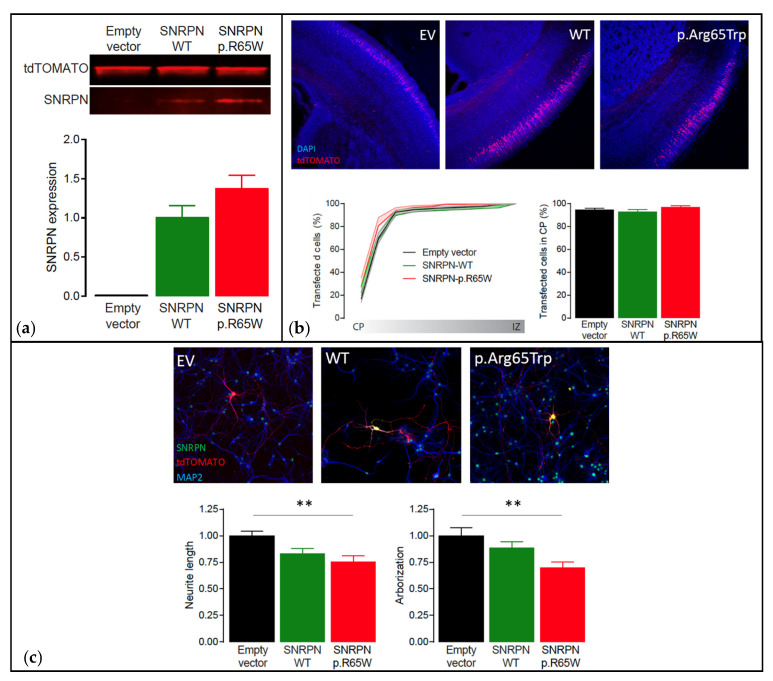
Functional analysis using the PR*i*SM screen. Abbreviations: Empty vector (EV), wild type (WT). (**a**) Western blot analysis of transfected HEK293T cells showing that *SNRPN* expression is normal. Top: Western blot of transfected HEK293T cells showing clear overexpression of *SNRPN-WT* and *SNRPN-p.R65W*. Bottom: Quantification of the Western blots showing that the *SNRPN-p.R65W* missense variant does not alter the expression levels of *SNRPN* (*N* = 4 per condition). (**b**) In utero electroporation of the different *SNRPN* constructs does not affect neuronal migration in vivo. Top: Representative images from in utero electroporated postnatal brains on day 1. These images show that the far majority of the transfected cells (tdTOMATO+) migrated out to the cortical plate (CP; the outer layer of the cortex). Bottom left: Cumulative distribution of the transfected neurons at P1 from the cortical plate (CP; the outer layer of the cortex) to the intermediate zone (IZ; inner layer of the cortex). Bottom right: Percentage of neurons that migrated out to the superficial layers of the cortex (sum of bins 1 through 4). Number of pictures/animals for each condition = 9/3. (**c**) Expression of the different *SNRPN* constructs in mouse primary hippocampal neurons reveal that overexpression of the p.Arg65Trp variant significantly affects neuronal maturation in vitro. Top: Representative images of primary hippocampal neurons transfected with empty vector control, *SNRPN-WT* or *SNRPN-p.Arg65Trp* (tdTOMATO+). Neurons are stained for SNRPN (green) and MAP2 (blue). Bottom: Quantitative analysis of the total neurite length and arborization in the different conditions. Number of neurons/number of batches for each condition = 20/2; ** *p* < 0.01.

Functional analysis showed that the NM_003097.3(*SNRPN*):c.193C>T, p.(Arg65Trp) variant did not cause instability of the protein, showing similar expression levels to *SNRPN-WT* (*p* = 0.16, two-tailed unpaired Student’s *t*-test) (Figure 5a). Additionally, overexpression of *SNRPN-WT* or *SNRPN-p.Arg65Trp* in a small subset of neurons during embryological development did not alter the migration pattern of the neurons transfected (One-Way ANOVA: F[2,29] = 0.97, *p* = 0.39) (Figure 5b). However, whereas overexpression of *SNRPN-WT* showed a small trend in reducing neuronal maturation (Neurite length: One-Way ANOVA: F[2,45] = 6.29, *p* = 0.003; *SNRPN-WT* versus empty vector control: *p* = 0.0627, Tukey’s multiple comparison test; Arborization: One-Way ANOVA: F[2,45] = 5.66, *p* = 0.006; *SNRPN-WT* versus empty vector control: *p* = 0.44, Tukey’s multiple comparison test), overexpression of the *SNRPN-p.Arg65Trp* variant significantly affected neuronal maturation in vitro, resulting in a decreased neurite length and arborization (Neurite length: *SNRPN-p.Arg65Trp* versus empty vector control: *p* = 0.003, Tukey’s multiple comparison test; Arborization: *SNRPN-p.Arg65Trp* versus empty vector control: *p* = 0.004, Tukey’s multiple comparison test) (Figure 5c). Taken together, these results suggest that the *SNRPN-p.Arg65Trp* variant does affect the function of SNRPN, suggesting pathogenicity.

### 3.2. Literature Review

**Figure 6:**
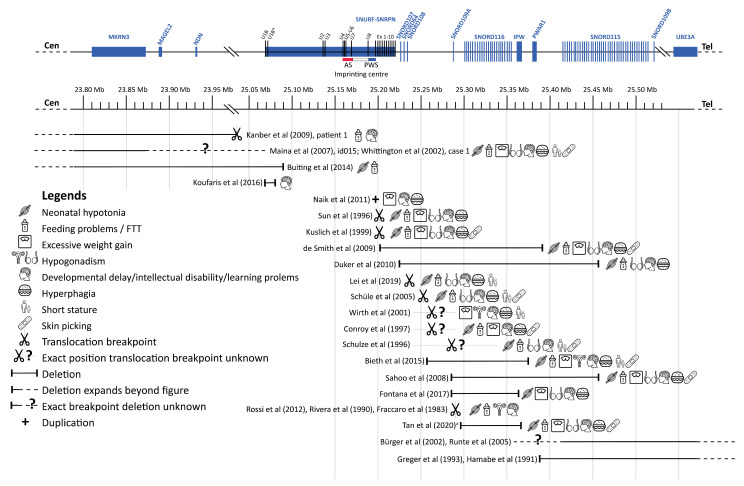
Overview cases literature review. Abbreviations: Angelman syndrome (AS), failure to thrive (FFT), Prader–Willi syndrome (PWS). ^a^ Exact breakpoints for this case are not available, depicted is the minimal deleted region. This figure shows an overview of the cases we found during our literature review. For each case, the affected region is depicted, followed by symbols representing the clinical phenotype of the case. Genes are represented at scale with physical distance in Mb. SnoRNAs are depicted as vertical lines and other genes as boxes. Deletions are depicted according to the genomic deletion coordinates for build hg19. Excessive weight gain was only scored as present if it occurred when the case was 1 to 6 years old. Figure adapted from Fontana et al. [19] and Tan et al. [13]. This figure has been designed using resources from Flaticon.com (from surang, freepik, smashicons, dinosoftlabs and monkik), accessed on 1 March 2021.

To interpret our findings in the light of the available literature, we performed a thorough literature search for case reports or case series of patients with genetic alterations (e.g., translocations, deletions, genetic variants) in the PWS critical region on the paternal chromosome that affected only part of the PWS critical region. After deduplication, we found 4693 articles, of which the titles and abstracts were reviewed. After full-text screening, we included 22 cases with PWLS. Additionally, we found 29 case reports that described abnormalities in only *MAGEL2* (associated with Schaaf–Yang syndrome) and 42 case reports with abnormalities in only *MKRN3* (associated with central precocious puberty), which are not included in the table. Most of the 22 cases with PWLS had a translocation or a deletion, leading to a loss of function of one or more genes of the PWS critical region. Additionally, we found one case report of a patient with a small duplication in exon 1 of *SNURF*, which introduced a premature stop codon in *SNURF*. The results of this literature review are summarized in Table 3 and Figure 6.

## 4. Discussion

We here present the puzzling diagnostic trajectory of a 46-year-old female patient with PWLS. After genetic testing excluded the classical PWS genetic defects (deletion, mUPD, ICD, or translocation of chromosome 15q11.2-13), we performed additional genetic analyses. Obesity gene panel analysis of 57 obesity-associated genes revealed a homozygous variant NM_003097.3(SNRPN):c.193C>T, p.(Arg65Trp) in exon 6 of the *SNURF-SNRPN* gene, changing CGG into TGG (Figure 2). This variant was not present in the 1000 Genomes Project (Phase 3 release) [42] or in the genome aggregation database (gnomAD v2.1.1) [43]. The variant changes the non-aromatic and positively charged arginine into the aromatic, non-polar tryptophan (Arg65Trp), which is deleterious according to various variant effect predictor programs. The fact that this homozygous missense variant is located in the PWS critical region suggests a relation with the PWS phenotype of the patient.

While the patient’s phenotype included PWS features, she also had atypical features, including brain lesions at MRI. Although morphological abnormalities in the brain of patients with PWS have been described [67,68,69,70,71], the brain lesions found in our patient were atypical for PWS. SNP array analysis showed multiple large ROH spread across different chromosomes, suggesting that (multiple) autosomal recessive diagnoses could contribute to the complete phenotype in this patient. Additionally, the fact that the mother of the patient had the same variant on her paternal chromosome 15 without having PWLS (she had obesity but did not fulfil any other diagnostic criteria for PWS), suggests that this variant is probably not the only explanation for the phenotype of the index case.

As she had dysgenesis of midline brain structures and optic nerve hypoplasia at MRI with hypothalamic-pituitary dysfunction [38], she fulfilled the criteria for SOD. SOD has a wide variability of clinical features, including some features that were present in our patient, like developmental delay [72], dysmorphic features, and autism-like behavior [73]. However, our patient also had many symptoms not associated with SOD, including hyperphagia, hypotonia, and kyphosis. Although the etiology remains unclear in most cases, SOD has been associated with pathogenic variants in *HESX1, OTX2, SOX2*, and *SOX3* [38,74,75]. One of the ROH of the index patient included *SOX3*, but none of the ROH included *HESX1*, *OTX2*, or *SOX2*.

The index case has a remarkable family history with a large pedigree. Most family members were short, overweight, and/or illiterate. The latter is remarkable in the context of 10% inadequate literacy in the total Dutch 16–65 year old population [76]. The high prevalence of short stature, obesity and/or illiteracy in this family is suggestive of a genetic component. Unfortunately, other family members were unavailable for genetic analysis.

Interestingly, *SNURF-SNRPN* is a bi-cistronic gene, which is transcribed exclusively from the paternally inherited chromosome. *SNURF-SNRPN* is expressed in several tissues and shows the highest RNA expression in the brain and heart, followed by endocrine tissues, male reproductive tissues, and blood [77]. *SNURF-SNRPN* encodes two proteins: SNURF and SNRPN (also called SmN) [78]. The function of SNURF is unknown [78]. SNRPN is involved in mRNA splicing and predominantly expressed in neurons, especially in central neurons [79,80,81]. SNRPN influences neurite outgrowth, neuron migration, and distribution of dendritic spines in mice [82]. However, the precise role of SNRPN in the development of the PWS phenotype remains largely unknown. Apart from encoding SNURF and SNRPN proteins, *SNURF-SNRPN* transcription is necessary for production of downstream ncRNAs implicated in many PWS traits [83,84].

Imprinting at 15q11.2-13 is regulated by a bipartite imprinting center defined as the smaller regions of overlap (SRO) found in patients with imprinting defects [85,86,87,88,89,90]. The Angelman syndrome-SRO (AS-SRO) functions to silence paternally expressed genes on the maternal allele. The PWS-SRO functions somatically to activate paternally expressed genes on the paternal allele [91,92,93]. The PWS-SRO spans *SNURF-SNRPN* exon 1 [94,95]. The missense variant in this patient is in exon 6, about 20 kb downstream of the PWS-SRO and is therefore very unlikely to generate an ICD. Additionally, in our patient, imprinting and expression of the genes in the PWS critical region was normal, excluding an ICD.

The PWS critical region encompasses several genes, including *MKRN3*, *MAGEL2*, *NDN*, *SNURF-SNRPN,* and ncRNAs including snoRNAs like *SNORD115* and *SNORD116* [10]. Two individuals with deletions of *MKRN3, MAGEL2,* and *NDN* have been described [60,64]. These patients lacked many typical PWS features, suggesting that deletions of these genes cannot cause the full PWS phenotype on their own. However, patients with a heterozygous truncating pathogenic variant in the paternally derived allele of the *MAGEL2* gene have Schaaf–Yang syndrome, which has clinical overlap with PWS, including intellectual disability, autism spectrum disorder, neonatal hypotonia, and infantile feeding problems [96,97]. Patients with Schaaf–Yang syndrome also display non-PWS features, for example distal joint contractures. *SNORD115* is unlikely to play a role in the PWS phenotype, as individuals with a paternally inherited deletion of *SNORD115* do not have PWS features [47,48,56,57]. *SNORD116* is suspected to be a strong contributor to the PWS phenotype [98]. Several cases with PWS features and microdeletions or translocations resulting in lack of expression of *SNORD116* and *IPW* indicate an important role of *SNORD116* and *IPW* [13,14,15,16,17,18,19,49,50,51,52,53,58,66], Table 3. However, *IPW* is a non-protein coding RNA, which is minimally conserved between human and mouse genomes [99,100]. Mice with a targeted deletion of *SNORD116* show cognitive deficits, abnormal growth and feeding [101,102], while mice with paternal deletions of portions of *SNURF* or *SNRPN* seem to have a normal phenotype [103,104]. It has been suggested that individuals with translocations have a milder phenotype when only *SNORD116* is affected, compared to individuals in whom *SNURF-SNRPN* expression is also abolished. *SNORD116* deletions seem to cause most of the major features of PWS, but they may be less prominent [58]. It should be noted that this is hard to investigate as all patients are assessed by different physicians, making it hard to objectively assess patients [84]. Lastly, *SNURF* could contribute to the PWS phenotype, as is demonstrated by Naik et al. [61], who presented a case with a small duplication in exon 1 of *SNURF-SNRPN*, which is predicted to only affect *SNURF* expression, although it cannot be ruled out that *SNRPN* is also affected. This patient had developmental delay, challenging behavior, and increased appetite, but also tall stature (170 cm (+3.26 SD)) and a large head circumference (57 cm (+2.69 SD)), which are not PWS features and were also not present in the index case that we present.

*SNORD116* is produced from the same primary transcript as *SNURF-SNRPN* [83,84] and therefore variants in *SNURF-SNRPN* could cause a PWS phenotype through a change of expression of *SNORD116*. However, the RNA sequencing in our patient showed a normal expression of *SNORD116*. This rules out that the PWLS was caused by lack of *SNORD116* expression.

PWS is generally thought to result from loss of expression of the paternal genes on the PWS critical region, as the maternal PWS critical region is imprinted and therefore not expressed. The mother of the index case had the same variant as the index case on her paternal chromosome 15. She did not have a documented intellectual disability or specific PWS features, but she was illiterate and had obesity. This suggests that the *SNRPN-p.Arg65Trp* variant might not always be pathogenic (incomplete penetrance) or might result in a mild phenotype. There is some evidence from mice that expression of the maternal allele of chromosome 15q11.2-13 can be detected when the paternal allele is not active [105,106]. However, findings in mice are poor predictors of the human situation and therefore the loss of silencing found by Rieusset et al. [105] cannot be extrapolated to humans.

A previous report by Iourov et al. [107] suggested that homozygosity (without mUPD) of part of 15q11.2 could be related to a mild PWLS phenotype, mainly characterized by developmental delay and/or intellectual disability. According to SNP array, homozygosity of 15q11.2 was also present in our patient and could have influenced her phenotype. However, the phenotype of our index case was specific to PWS, whereas the five cases described by Iourov et al. lacked most major phenotypic features of PWS. They did not present hypothalamic dysfunction leading to hyperphagia, pituitary hormone deficiencies, abnormal temperature regulation, or inadequate pain registration.

In our patient, RNA sequencing showed that expression of *SNURF-SNRPN* and the other genes in the PWS critical region were not affected. However, the change from arginine to tryptophan caused by the homozygous *SNRPN* variant was predicted to severely affect protein function. In order to understand whether the variant found in this patient indeed is pathogenic and whether it causes a loss of function or a gain of function, we made use of the unbiased functional genomics screen PR*i*SM. This showed that the variant does not cause protein instability, nor behaves differently from *SNRPN-WT* in the migration assay. However, whereas overexpression of *SNRPN-WT* resulted in a small but non-significant trend in reduced neural development in vitro, *SNRPN-p.Arg65Trp* significantly reduced neural development upon overexpression primary hippocampal neurons. This suggests that the missense variant might alter the function of SNRPN, causing a dominant negative effect on neuronal maturation when overexpressed. These results show that this variant does not cause a loss of function, rather it might suggest that it causes a gain of function. However, future studies are needed to confirm its functional impact.

The *SNRPB’/B* genes encode the proteins small nuclear ribonucleoprotein-associated polypeptide B’ and B (SmB’/B). They are closely related to *SNRPN*, which encodes the protein SNRPN, also called small nuclear ribonucleoprotein-associated polypeptide N (SmN). Normally, SNRPN replaces SmB’/B in the brain. It has been demonstrated that the loss of SNRPN in PWS brain tissue causes a compensatory feedback loop that drastically upregulates the levels of SmB’/B. It has been suggested that upregulation of SmB’/B in PWS reduces the severity of the syndrome [108]. However, in the presence of a non-functional SNRPN protein, this compensation mechanism might not be induced, leading to more severe phenotypic features compared to cases where SNRPN is completely absent. RNA sequencing in this patient showed that *SNRPB’/B* expression in fibroblasts was normal.

## 5. Conclusions

In conclusion, the finding of this homozygous missense *SNURF-SNRPN* variant in a patient with virtually all clinical features of PWS suggests that this variant might have caused her PWLS with combined pituitary hormone deficiency (CPHD). Additionally, functional analysis suggested that the variant might affect the function of SNRPN. However, the large ROHs that were found throughout the genome suggests other autosomal recessive conditions that could contribute to her complete phenotype. The fascinating trajectory of genetic and functional analyses performed in this patient shows that the finding of a unique variant in the PWS critical region, in a patient with PWLS, does not necessarily prove a causal relationship between the two.

## Figures and Tables

**Table 1:** Consensus criteria Prader–Willi syndrome and the score of the index case.

Major Criteria (1 Point Each)	Score	Minor Criteria (0.5 Points Each)	Score
Neonatal/infantile hypotonia and poor suck	**1**	Decreased foetal movement and infantile lethargy	**0.5**
Feeding problems and failure to thrive as infant	**1**	Typical behaviour problems	**0.5**
Weight gain at 1–6 years; obesity; hyperphagia	**1**	Sleep apnoea	**0.5**
Characteristic dysmorphic facial features	**1**	Short stature	**0**
Hypogonadism with small genitalia, pubertal delay and insufficiency	**1**	Hypopigmentation	**0**
		Small hands and feet	**0.5**
Developmental delay/intellectual disability	**1**	Narrow hands, straight ulnar border	**0.5**
Deletion or other cytogenetic/molecular abnormality of the Prader-Willi chromosome region, including maternal disomy	**0**	Esotropia, myopia	**0.5**
		Thick, viscous saliva	**0**
		Speech articulation defects	**0.5**
		Skin picking	**0.5**
**Total major**	**6**	**Total minor**	**4**
**Total points index case**	**10 points**		
**Requirements clinical diagnosis PWS for adults**	**8 points, of which 5 points major**		

Major criteria are weighted at one point each and minor criteria at half a point each. For adults, a total score of eight, of which at least five points for the major criteria, is necessary for the clinical diagnosis of PWS [8].

**Table 2:** Clinical features, dysmorphic features, and challenging behavior associated with PWS and presence in the index case.

Clinical Features [1,2,3,5,6,39,40,41]	Present in Index Case	Prevalent in PWS
Poor foetal movement	yes	yes
Hypotonia during infancy	yes	yes
Feeding problems during infancy	yes	yes
Abnormal pubertal development	yes	yes
Developmental delay Speech Psychomotor	yes yes	yes yes
Intellectual disability	yes	yes
Sleep related breathing disorder	yes	yes
Osteoporosis	yes	yes
Diabetes mellitus type 2	yes	yes
Hypertension	no	yes
Abnormal pain registration	no	yes
Pituitary hormone deficiencies Hypogonadism Growth hormone deficiency Hypothyroidism	yes yes no	yes yes yes
Vitamin D deficiency	yes	yes
Bowel problems Obstipation	yes	yes
Abnormal temperature regulation	no	yes
Leg edema	no	yes
Foot problems	yes	yes
Challenging behaviour Skin picking Hair pulling Temper tantrums Stealing food	yes yes yes yes	yes yes yes yes
Obesity	yes	yes
Hyperphagia	yes	yes
Dysmorphic features Narrow temple distance Narrow nasal bridge Almond-shaped eyes Small palpebral fissure length Strabismus Thin upper lip Low hair line Small chin Broad nose Small hands and feet Scoliosis Kyphosis	yes no yes yes yes no yes yes yes yes no yes	yes yes yes no yes yes no no no yes yes yes
Short stature (height below −2 SD)	no	yes

Abbreviations: IGF-1, insulin-like growth factor 1; GH, growth hormone; SD, standard deviation.

**Table 3:** Literature review.

Case and Reference	Sensitivity [46]	Greger et al. (1993) & Hamabe et al. (1991) [47,48]	Schulze et al. (1996) [49]	Sun et al. (1996) [50]	Conroy et al. (1997) [51]	Kuslich et al. (1999) [52]	Wirth et al. (2001) [53]
**Genetic abnormality**		1.5 Mb deletion paternally transmitted	Balanced translocation t(9;15)(q21;q12–13)	Balanced translocation t(15;19)(q12;q13.41)	Balanced translocation t(2;15)(q37.2;q11.2)	Balanced translocation t(4;15)(q27;q11.2)	Balanced translocation t(X;15)(q28;q12)
**Location**		*SNORD115* to *GABRB3* intron 3	*SNRPN* exon 20/intron 20 (located between *SNORD108* and *SNORD109A*)	*SNRPN* intron 2	*SNRPN* exon 20/intron 20 (located between *SNORD108* and *SNORD109A*)	*SNRPN* intron 2	*SNRPN* exon 20/intron 20 (located between *SNORD108* and *SNORD109A*)
**Gender**		3 family members	male	male	male	male	female
**Age at last examination (years)**			29	3	4	11	20
**Weight (kg)**			89	34	>95th percentile	NA	72
**Height (cm)**			169	103	50–75th percentile	NA	151
**Major criteria** Neonatal hypotonia Feeding problems/FTT Excessive weight gain 1–6 years Characteristic facial features Hypogonadism/genital hypoplasia/delayed or incomplete puberty Developmental delay/intellectual disability/learning problems Hyperphagia/food foraging/obsession with food	98 96 95 49 96 98 93	no phenotype when paternally transmitted (causes Angelman syndrome when maternally transmitted)	+ + - (at 7 years) + + + NA	+ + + + + + +	+ + + + NA + +	+ + + + + + +	- - + - + + +
**Minor criteria** Decreased foetal movements/infantile lethargy Challenging behaviour Sleep disturbance or sleep apnoea Short stature Hypopigmentation Small hands/feet for height and age Narrow hand, straight ulnar border Eye abnormalities (esotropia, myopia) Thick, viscous saliva Speech articulation defects Skin picking	89 82 37 86 47 75 69 49 83 93 61		NA + + + + + NA + NA - +	+ + - - - + NA - - - -	+ + NA − NA − NA + + + +	+ + + NA − − − − + + +	− + − + − − NA + NA − −
**Case and Reference**		**Maina et al. (2007) id05 and Whittington et al. (2002) Case 1** [54,55]	**Bürger et al. (2002) & Runte et al. (2005)** [56,57]	**Schüle et al. (2005)** [58]	**Sahoo et al. (2008)** [14]	**Calounova et al. (2008) patient 2** [59]	**de Smith et al. (2009)** [15]
**Genetic abnormality**		Deletion of maximum 1.8 Mb	~570 kb deletion paternally transmitted	Balanced translocation t(4;15)(q27;11.2)	175 kb deletion	9.5 Mb deletion	187 kb deletion
**Location**		Includes: *CYFIP1* and *MKRN3*, but not *SNRPN*	*SNORD115* UTAI *UBE3A*	*SNRPN* intron 17 (=between *SNORD108* and *SNORD109A*)	*SNORD109A* UTAI *SNORD115-24*	*NPAP1* UTAI *AVEN*	*SNURF-SNRPN* exon 2 UTAI *IPW*
**Gender**		male	3 family members	male	male	female	male
**Age at last examination (years)**		22		22	4	18	19
**Weight (kg)**		NA		90	63	79	109
**Height (cm)**		NA		164	115	149	167.5
**Major criteria** Neonatal hypotonia Feeding problems/FTT Excessive weight gain 1–6 years Characteristic facial features Hypogonadism/genital hypoplasia/delayed or incomplete puberty Developmental delay/intellectual disability/ learning problems Hyperphagia/food foraging/obsession with food		+ + + NA + + +	no phenotype when paternally transmitted (causes Angelman syndrome when maternally transmitted)	+ + −(at 8 years) − + +/− +	+ + + + + + +	+ + NA NA + + NA	+ + + +/− + + +
**Minor criteria** Decreased foetal movements/infantile lethargy Challenging behaviour Sleep disturbance or sleep apnoea Short stature Hypopigmentation Small hands/feet for height and age Narrow hand, straight ulnar border Eye abnormalities (esotropia, myopia) Thick, viscous saliva Speech articulation defects Skin picking		+ + + + + + NA + + + +		+ + + + − + − + − − +	+ + + − NA + NA − − + +	NA + NA + NA NA NA + NA NA NA	NA + NA − NA + NA NA NA NA +
**Case and Reference**		**Patient 1 in Kanber et al. (2009)** [60]	**Duker et al. (2010)** [16]	**Naik et al. (2011)** [61]	**Rossi et al. (2012) & Rivera et al. (1990) & Fraccaro et al. (1983)** [45,62,63]	**Buiting et al. (2014)** [64]	**Bieth et al. (2015)** [17]
**Genetic abnormality**		Unbalanced translocation t(X;15)(q28;q11.2)	236 kb deletion	25 bp duplication	Jumping translocation with a major t(15;18)(q13;q23) and a minor t(X;15)(q28;q13) cell line	~3.9 Mb deletion	118 kb deletion
**Location**		Breakpoint: between *NDN* and *SNURF-SNRPN* Deletion: *CYFIP1* UTAI *NDN*	3′ end of *SNRPN* (exon 10) UTAI *SNORD155-24*	Exon 1 *SNURF/SNRPN*	Between *SNORD108* & *SNORD116*	*CHEK2P2* UTAI *SNRPN* exon U1B*	*SNORD109A* UTAI *IPW*
**Gender**		female	male	female	female	male	female
**Age at last examination (years)**		12	11	11	10 months	3	23
**Weight (kg)**		75	94	63.5	9.9	17.3	75
**Height (cm)**		160	10–25th percentile	170	70	103	155 (6m GHT)
**Major criteria** Neonatal hypotonia Feeding problems/FTT Excessive weight gain 1–6 years Characteristic facial features Hypogonadism/genital hypoplasia/delayed or incomplete puberty Developmental delay/intellectual disability/learning problems Hyperphagia/food foraging/obsession with food		− + NA − − + −	+ + −(7 months) − + + +	− − + − NA + +/−	+ + NA + + + NA	Asymptomatic apart from delayed motor skills and transient muscular hypotonia associated with mild feeding difficulties in infancy	+ + + + + + +
**Minor criteria** Decreased foetal movements/infantile lethargy Challenging behaviour Sleep disturbance or sleep apnoea Short stature Hypopigmentation Small hands/feet for height and age Narrow hand, straight ulnar border Eye abnormalities (esotropia, myopia) Thick, viscous saliva Speech articulation defects Skin picking		− − − − − − NA NA − − −	+ + + NA − − NA + + + −	− + NA − NA NA NA NA NA NA NA	+ NA NA NA + NA NA NA NA NA NA		NA + + + NA + NA + + NA +
**Case and Reference**		**Koufaris et al. (2016)** [65]		**Fontana et al. (2017)** [19]		**Lei et al. (2019)** [66]	**Tan et al. (2020)** [13]
**Genetic abnormality**		13 kb deletion		~ 81 kb deletion		Balanced translocation t(15;19)(q11.2;q13.3)	71 kb deletion
**Location**		U1B and U1B* upstream exons of *SNRPN*		*SNORD109A* to exon 3 *IPW*		Between *PAR5* (including *SNORD108*) and *PAR6*	At least *SNORD116* and *IPW*
**Gender**		NA		male		male	male
**Age at last examination (years)**		child		18		13	17
**Weight (kg)**		overweight		76		45	BMI: 28.45 kg/m^2^
**Height (cm)**		NA		174		132	181
**Major criteria** Neonatal hypotonia Feeding problems/FTT Excessive weight gain 1-6 years Characteristic facial features Hypogonadism/genital hypoplasia/delayed or incomplete puberty Developmental delay/intellectual disability/ learning problems Hyperphagia/food foraging/obsession with food		Overweight child with mild intellectual disability and neurodevelopmental delay		+ NA + − + +/− +		+ + − (7 months) NA + + +	+ + + + − + +
**Minor criteria** Decreased foetal movements/infantile lethargy Challenging behaviour Sleep disturbance or sleep apnoea Short stature Hypopigmentation Small hands/feet for height and age Narrow hand, straight ulnar border Eye abnormalities (esotropia, myopia) Thick, viscous saliva Speech articulation defects Skin picking				NA NA NA − NA − NA + NA NA NA		+ + − + − − − − − − −	+ + + − NA − NA − NA NA +

Excessive weight gain was only scored as present if it occurred when the case was 1 to 6 years old. Challenging behavior was scored as ‘+’ when at least one typical challenging behavior was present (temper tantrums, violent outbursts and obsessive/compulsive behavior; tendency to be argumentative, oppositional, rigid, manipulative, possessive, and stubborn; perseverating, stealing, and lying). Abbreviations: Body mass index (BMI), growth hormone therapy (GHT), failure to thrive (FTT), up to and including (UTAI), +: present, −: absent, +/−: somewhat present, N/A: not described, /: and/or.

## Data Availability

Not applicable.

## Supplementary Materials

The following are available online at https://www.mdpi.com/article/10.3390/genes12060875/s1, Table S1: Endocrine evaluations of the index case, Table S2: Full search strategy (Embase), Table S3: Regions of homozygosity detected by SNP array.
