# Supplementary material for: The Diagnostic Journey of a Patient with Prader–Willi-Like Syndrome and a Unique Homozygous SNURF-SNRPN Variant; Bio-Molecular Analysis and Review of the Literature
Source: Genes (Basel). 2021 Jun 7;12(6):875. doi: 10.3390/genes12060875 (PMC8227738; doi:10.3390/genes12060875)
Supplement: Supplementary file 1 [file genes-12-00875-s001.zip › genes-1223292-supplementary.pdf]

Supplementary data

Legends

Table S1. Endocrine evaluations of the index case

Table S2. Full search strategy (Embase)

Table S3. Regions of homozygosity (ROH) detected by SNP array

Table S1. Endocrine evaluations of the index case

	Reference range	Value
LH (U/L)	2 – 55	1.0
FSH (U/L)	1 – 15	2.1
Oestradiol (pmol/L)	55 – 1285	76
TSH (mU/L)	0.4 – 4.3	1.28
Free T4 (pmol/L)	11 – 25	16.7
Cortisol (nmol/L)	200 – 700	320
IGF-1 (nmol/L)	6.8 – 26.5	5.1
GH peak (µg/L) *	>4.2	2.9
Prolactin (U/L)	0.1 – 0.98	0.11
SHBG (nmol/L)	20 – 120	18
25-OH vit D (nmol/L)	50 – 120	15
Glucose (mmol/L)	4 – 6.1	6.2
HbA1c (mmol/mol)	26 – 42	50

Medication at time of evaluation: psyllium (laxative drug). Abbreviations: TSH, thyroid-stimulating hormone; LH, luteinizing hormone; FSH, follicle stimulating hormone; IGF-1, insulin-like growth factor 1; SHBG, sex hormone binding globulin; 25-OH vit D, 25-hydroxy vitamin D; HbA1c, haemoglobin A1c.

*GH peak during growth hormone releasing hormone (GHRH) plus arginine stimulation test.

Table S2. Full search strategy (Embase)

(((Prader-willi-like):ab,ti,kw) OR (('Prader Willi syndrome'/exp OR 'mkrn3 gene'/de OR 'makorin ring finger protein 3'/de OR 'magel2 gene'/de OR 'magel2 protein'/de OR 'necdin'/de OR 'small nuclear ribonucleoprotein polypeptide n'/de OR 'snrpn gene'/de OR 'snrpn protein'/de OR 'e6 associated protein'/de OR ((Prader* NEAR/3 Willi*) OR PraderWilli OR 15q11* OR mkrn3 OR makorin-3 OR znf127 OR D15S9 OR RNF63 OR EC-2.3.2.27 OR ZFP127 OR CPPB2 OR magel2 OR magel-2 OR ((makorin) NEAR/3 (ring) NEAR/3 (3)) OR ((zinc) NEAR/3 (finger) NEAR/3 (127)) OR ((ring) NEAR/3 (finger) NEAR/3 (63)) OR ((MAGE OR melanoma) NEAR/3 (family) NEAR/3 (L2)) OR ((necdin-like OR MAGE-like) NEAR/3 (protein) NEAR/3 (1 OR 2)) OR NDNL1 OR MAGE-Like-2 OR SHFYNG OR PWLS OR NM15 OR (NDN NOT nonclassic-differentiation-number*) OR necdin* OR HsT16328 OR PWCR OR C15orf2 OR NPAP1 OR NPAP-1 OR Nuclear-Pore-Associated-Protein-1 OR ((chromosome-15) NEAR/3 (open-reading-frame* OR ORF*) NEAR/3 (2)) OR SNURF-SNRPN* OR SNRPN* OR Small-Nuclear-Ribonucleoprotein-Polypeptide-N* OR Small-Nuclear-Ribonucleoprotein-Associated-Protein-N* OR Tissue-Specific-Splicing-Protein* OR Sm-Protein-D OR SM-Protein-N OR HCERN3 OR (((Sm-N OR SMN OR Sm-D OR PET1 OR BEY OR PED) AND (gene OR genes OR genetic OR genome* OR genomic)) NOT (sec*-malign*-neoplas* OR survival-motor-neuro*)) OR Small-Nucl*-Ribonucleoprot*-N OR RT-LI OR PWCR OR SNORD107 OR SNORD-107 OR (("C/D" OR CD) NEAR/3 (box*) NEAR/3 (107 OR 64 OR 109A OR 116 OR 115 OR 109B)) OR HBII-436 OR RF01164 OR SNORD64 OR HBII-13 OR RF00570 OR SNORD109A OR HBII-438A OR RF01278 OR SNORD116 OR HBII-85 OR PWCR1 OR SNORD115 OR HBII-52 OR RNHBII52 OR RF00105 OR SNORD109B OR HBII-438B OR RF01278 OR KIAA1899):ab,ti,kw) AND ('case report'/de OR 'case study'/exp OR (case* OR patient OR man OR woman OR famil* OR male OR female OR boy* OR girl* OR subject* OR child OR adult OR sib*):ab,ti,kw) AND ('gene mutation'/exp OR 'DNA methylation'/de OR 'methylation'/de OR (mutat* OR delet* OR insert* OR loss* OR disrupt* OR variant* OR translocate* OR phenotyp* OR genotyp* OR monosom* OR disom* OR missens* OR nonsense* OR frameshift* OR repeat-expans* OR micordelet* OR pathogenes* OR methylat* OR alterat* OR invers* OR duplicat* OR aneuploid* OR ((gene* OR genetic* OR chromosom* OR allelic* OR splic* OR pathogen*) NEAR/3 (imbalanc* OR abnormal* OR defect* OR alter*))):ab,ti,kw))) NOT ([Conference Abstract]/lim) NOT ((animal/exp OR animal*:de OR nonhuman/de) NOT ('human'/exp))

The search strategy was adjusted, but the same search terms were used for the databases Medline ALL, Web of Science Core Collection, and the Cochrane Central Register of Controlled Trials.

Table S3. Regions of homozygosity (ROH) detected by SNP array

Chromosome	Start (Mb)	End (Mb)
**chromosome 1**	0	14079992
	17754824	23910356
	30986712	38689117
	48858721	86362747
	203496639	244933063
**chromosome 2**	16276659	46042578
	112092902	121691091
	146091570	173000514
	217204152	241088419
**chromosome 3**	0	7359545
	134562583	169721188
**chromosome 4**	20432457	24391806
	108645163	137649082
**chromosome 5**	0	8499922
	70260650	75783963
**chromosome 6**	7400923	52530496
	52661586	91488615
	120833805	145389697
**chromosome 7**	30761964	57793473
	61078136	74883098
**chromosome 8**	8139051	19144793
**chromosome 9**	106785682	131335664
**chromosome 10**	0	47071299
	47209126	55547845
	99630008	135374737
**chromosome 12**	127206952	132349534
**chromosome 13**	106829225	109514411
**chromosome 14**	19492651	32980643
	90156855	106368585
**chromosome 15**	21668641	25424023
**chromosome 17**	2573236	14584456
	42298976	73819072
**chromosome 18**	3983327	11214052
	26339697	59116707
	62278132	76117153
**chromosome 19**	54566177	59259139
**chromosome 20**	248587	8113380
	17217266	49522932
	49865623	62435964
**chromosome 21**	18063432	27311531
**chromosome 22**	35673068	41890350
**chromosome X**	5216582	15177653

Start and end positions of the ROH are given for build hg18.
